# Astragaloside IV ameliorates atrazine-induced male reproductive toxicity: an *in vivo* and *in silico* analysis

**DOI:** 10.3389/ftox.2025.1692518

**Published:** 2025-12-22

**Authors:** Srinivasa Rao Sirasanagandla, Mohamed Al Mushaiqri, Firas Al-Majrafi, Nadia Al-Abri, Selvaraj Jayaraman, Isehaq Saif Al Huseini

**Affiliations:** 1 Department of Human and Clinical Anatomy, College of Medicine and Health Sciences, Sultan Qaboos University, Muscat, Seeb, Oman; 2 College of Medicine and Health Sciences, Sultan Qaboos University, Muscat, Seeb, Oman; 3 Department of Pathology, College of Medicine and Health Sciences, Sultan Qaboos University, Muscat, Seeb, Oman; 4 Centre of Molecular Medicine and Diagnostics (COMManD), Department of Biochemistry, Saveetha Dental College and Hospitals, Saveetha Institute of Medical and Technical Sciences, Saveetha University, Chennai, India; 5 Department of Physiology, College of Medicine and Health Sciences, Sultan Qaboos University, Muscat, Seeb, Oman

**Keywords:** atrazine, astragaloside IV, reproduction, toxicity, herbicide, pollution

## Abstract

**Introduction:**

Atrazine (ATZ) stands as the most widely utilized herbicide globally and is known for its adverse impacts on the reproductive system. Although astragaloside IV (AS IV) is well known for possessing various health benefits, its protective effects against ATZ-induced toxicity remain unexplored. This study aimed to investigate the ameliorative potential of AS IV against ATZ-induced male reproductive toxicity in mice.

**Methods:**

Eight-week-old CD-1 mice were allocated into four groups (n = 10). ATZ and AS IV were administered at doses of 100 mg/kg/day and 40 mg/kg/day, respectively. Treatments were continued for 21 days, after which the animals were sacrificed for plasma biochemical analyses and testes collection for histopathological examination. One-way analysis of variance (ANOVA) followed by Bonferroni’s multiple comparison test was used for data analysis. Molecular docking studies were performed to evaluate ATZ and AS IV interactions with oxidative stress- and inflammation-related proteins, including glutathione (GSH), glutathione peroxidase (GPx), superoxide dismutase (SOD), and Nrf2, NF-κβ, IL-1β, IL-6, TNF-α, cullin-3, and Keap-1.

**Results:**

Biochemical analysis revealed significant reductions in GSH levels (*p < 0.001*), SOD activity (*p < 0.001*), and GPx activity (*p < 0.05*), along with elevated malonaldehyde levels (*p < 0.01*), following ATZ exposure. AS IV treatment in ATZ-exposed mice significantly improved these markers (*p < 0.05*). ATZ exposure led to significant decreases in testosterone (*p < 0.001*) and androgen-binding protein (ABP) levels (*p < 0.001*) within the ATZ group, whereas AS IV supplementation significantly improved these markers (*p < 0.05*). Histopathological examination revealed sloughed and collapsed seminiferous epithelia with vacuoles and poorly formed spermatids in ATZ-exposed mice, which were mitigated by AS IV treatment. The docking study revealed ATZ’s moderate interactions with key oxidative stress and inflammation-related proteins (binding energies: −4.7 to −5.5 kcal/mol), with glutathione (GSH) (−5.5 kcal/mol) showing the strongest binding. Notable stabilizations include SOD (three hydrogen bonds) and modulation of antioxidant (SOD, Nrf2) and anti-inflammatory (IL-1β and TNF-α) pathways. Moreover, AS IV demonstrated significant binding interactions with GSH (−9.2 kcal/mol), cullin-3 (−9.1 kcal/mol), and keap-1 (−8.9 kcal/mol). Molecular dynamics (MD) simulations showed strong stability for GPx and IL-1β targets against ATZ, and AS IV exhibited strong stability for GSH and cullin-3.

**Conclusion:**

AS IV appears to be a promising natural compound for preventing ATZ-induced male reproductive toxicity. Further investigations to elucidate the molecular mechanisms behind such positive effects are warranted.

## 1 Introduction

Herbicides, or weed killers, are a group of pesticides that are widely used in both agricultural and non-agricultural applications to control weeds. Their use is essential for increasing the yield of farm products by eradicating undesired weeds from crops (Mohd Ghazi et al., 2023; Hongoeb et al., 2025). However, widespread use of these compounds can adversely affect human health, leading to both mortality and morbidity (Hongoeb et al., 2025). Atrazine (ATZ) is among the most widely used herbicides worldwide, sold and used in agricultural applications in more than 100 countries (Li et al., 2024). ATZ is available as a white or colorless crystalline, odorless powder. It is slightly volatile, flammable, and chemically reactive. It is a chlorinated synthetic herbicide consisting of a triazine ring with a structural formula of 2-chloro-4-ethylamino-6-isopropylamino-1,3,5-triazine (C_8_H_14_ClN_5_) (Pathak and Dikshit, 2012). ATZ is frequently used to control weeds in commercial crops such as corn and sorghum, as well as fruits and vegetables (Nwani et al., 2010). It can enter the soil, water, and air through its use in various formulations, such as liquid sprays or granules, and it often contaminates natural water sources, including ponds, wells, and drinking water supplies (Bhatti et al., 2022). The half-life of ATZ varies across media: approximately 14 h in air (Rohr and Crumrine, 2005), less than 200 days in water, and 14–109 days in soil (Toxicological Profile for Atrazine, 2003).

Humans are exposed to ATZ through inhalation or ingestion of contaminated food, water, or soil. Once absorbed, ATZ is metabolized into various compounds and can accumulate in multiple organs (Hongoeb et al., 2025). ATZ, being an endocrine-disrupting chemical, can alter the hormonal signaling pathways and interfere with the hypothalamic–pituitary axis (Kucka et al., 2012). Evidence from a prospective cohort study demonstrated a potential association between the occupational ATZ exposure and the risk of prostate and lung cancers (Remigio et al., 2024). Experimental studies have demonstrated that ATZ exposure induces a wide range of adverse health effects. In rats, ATZ exposure has been shown to reduce basal metabolic rate, increase intra-abdominal fat deposition, cause ultrastructural changes in skeletal muscle and liver, and promote the toxicity of reproductive, neuro, and hepatorenal systems (Lim et al., 2009; Das et al., 2023; Zhao et al., 2024). Oral ATZ administration in rats has been found to induce salivary gland damage by triggering oxidative stress and promoting apoptosis (Ahmed et al., 2022). ATZ has been shown to induce cardiotoxicity by altering plasma lipid profiles, including total cholesterol, HDL-cholesterol, LDL-cholesterol, and triglycerides (Olayinka et al., 2022). In reproductive studies, ATZ exposure disrupted the blood–testis barrier proteins claudin-11 and connexin-43, thereby affecting spermatocytes and reducing the number of spermatids in the culture model of seminiferous tubules (Durand et al., 2020). Long-term exposure to ATZ has been linked to permanent testicular and seminiferous tubule atrophy and Leydig cell damage (Martins-Santos et al., 2017). Inhalation studies in mice have revealed that ATZ aerosol induces oxidative and nitrosative stress, increases cytokine production, lipid peroxidation, and apoptosis, and enhances mucus production and mast cell degranulation (Genovese et al., 2021).

Astragalosides (AS) are a group of major bioactive compounds present in the traditional Chinese medicine astragalus, or *Astragalus membranaceus* (Liang et al., 2023). Astragalosides are classified into four categories, AS I to AS IV, based on their structural differences. AS IV is a widely studied and biologically active compound due to its ability to modulate various cellular signaling pathways (Zhang et al., 2020; Yao et al., 2023). AS IV is a tetracyclic triterpenoid saponin derived from lanolin ester alcohol, available in white or yellow powder. AS IV is poorly soluble in water but readily soluble in organic solvents (Chen et al., 2021). Recent studies on astragalus derivatives demonstrated various pharmacological activities (Liang et al., 2023; Stępnik et al., 2025). In particular, AS IV exhibits multiple pharmacological effects that are mediated through the modulation of different signaling pathways, primarily antioxidant and anti-inflammatory pathways (Qi et al., 2017; Wang et al., 2019; Liang et al., 2023). Growing evidence suggests that AS IV can be used as a multi-target therapeutic agent (Brouns and De Deyn, 2009; Stępnik et al., 2025). A trans-membrane migration method was used to screen 18 types of Chinese herbs, of which only *Astragalus membranaceus* (AM) aqueous extract significantly stimulated human sperm motility *in vitro* (Hong et al., 1992). Another study showed that *in vitro* incubation with AM aqueous extract enhanced sperm motility in 30 infertile male volunteers (Liu et al., 2004). Additionally, AS IV was effective against bisphenol A-induced toxicity (Essawy et al., 2021). In other studies, AS IV supplementation significantly prevented cadmium-induced nephrotoxicity and male reproductive toxicity (Ning et al., 2022; Li et al., 2025). Though AS IV is well known to possess various health benefits through its antioxidant, anti-apoptotic, and anti-inflammatory properties, its potential therapeutic effects against ATZ-induced toxicity have not been reported. Hence, we aimed to study the ameliorative potential of AS IV against ATZ-induced male reproductive toxicity through *in vivo* and *in silico* analysis.

## 2 Materials and methods

### 2.1 Experimental animals

Eight-week-old CD-1 mice were used in the present study. Mice were allowed to acclimatize for 1 week before the start of the experiment. Mice were randomly divided into four equal groups, with each group consisting of ten animals (n = 10). The groups were a vehicle control group (CON), an ATZ group, an AS IV group, and an ATZ + AS IV group. They were housed in a controlled environment (12 h light/12 h dark cycle; room temperature 21 °C; 60% humidity) with free access to food (Oman Mills) and water. All animal experiments were conducted in conformity with the institutional guidelines and the international laws and policies (EEC Council directive 86/609, OJL 358, December 12, 1987; National Institutes of Health (NIH) Guide for the Care and Use of Laboratory Animals, NIH Publications No. 85-23, 1985). Body weight was recorded daily from the beginning to the end of the experiment. Ethical approval for the present study was obtained from the Sultan Qaboos University (SQU) Animal Ethics Committee (SQU/EC-AUR/2022-2023/2).

### 2.2 Dosage and treatments

ATZ (purity: 99.26%; MedChemExpress, United States) and AS IV (purity: ≥98.0%; MedChemExpress, United States) were used in this study. Mice in the ATZ, AS IV, and ATZ + AS IV groups were administered the compounds via oral gavage at doses of 100 mg/kg/day and 40 mg/kg/day, respectively. These doses were selected based on previous studies (Gely-Pernot et al., 2017; Li et al., 2025). The compounds were suspended with 0.5% carboxymethyl cellulose (CMC) for oral administration, and mice in the control (CON) group received 0.5% CMC solution only. Sterile conditions were maintained while preparing the solutions and giving the treatments. The treatments were given once daily and continued for 21 consecutive days. The treatment duration of 21 days was selected based on both toxicokinetic considerations of ATZ and previous experimental evidence demonstrating that a 3-week exposure period is sufficient to induce measurable reproductive toxicity and oxidative stress in rodents. ATZ is known to bioaccumulate in various tissues, including the testes, and exerts its toxic effects progressively through disruption of endocrine function, oxidative imbalance, and histopathological alterations. Studies have reported that subchronic exposure to ATZ for 14–28 days at doses ranging from 50 mg/kg/day to 100 mg/kg/day significantly alters reproductive hormone levels, sperm parameters, and antioxidant enzyme activity (Trentacoste et al., 2001; Jin et al., 2013; Martins-Santos et al., 2017). Therefore, a 21-day exposure period with 100 mg/kg/day represents an optimal duration to induce consistent and reproducible toxic effects without causing excessive systemic mortality or distress to the animals.

AS IV simultaneous supplementation allows adequate time for its protective pharmacological actions, including antioxidant, anti-inflammatory, and cytoprotective effects, to manifest. Several studies evaluating the protective effects of AS IV against toxicant- or drug-induced organ damage have also employed treatment durations of 3–4 weeks to effectively assess its biochemical and histological benefits (Ju et al., 2019; Hu et al., 2022; Li et al., 2025). Many studies reported that a lower dose of AS IV (40 mg/kg) was effective against various experimentally induced diseases (Ju et al., 2019; Lin et al., 2019; Hu et al., 2022; Zhang et al., 2023; Guo et al., 2024). Hence, the 21-day duration in this study was scientifically justified to ensure (i) sufficient ATZ-induced reproductive toxicity for evaluation and (ii) adequate time for AS IV to exert measurable ameliorative effects on biochemical and histopathological parameters. After the experimental period, mice were euthanized by an intraperitoneal injection of 70 mg/kg ketamine and 10 mg/kg xylazine. Blood and right and left testicles were collected for biochemical analysis, histopathology, and transmission electron microscopy.

### 2.3 Blood collection and biochemical analysis

After 24 h following the last dose, each animal was anesthetized by intraperitoneal injection of ketamine and xylazine. Blood was collected from the inferior vena cava into sterilized microfuge tube containing anticoagulant. The plasma was obtained after centrifugation and stored at −80 °C for biochemical analysis. The biochemical measurement of testosterone and androgen-binding protein (ABP) was carried out by mouse-specific enzyme-linked immunosorbent assay (ELISA) kits as per the manufacturer’s instructions of commercially available kits (Shanghai Sunred Biological Technology, Shanghai, China). The liver was dissected carefully, and approximately 150 mg of liver tissue was collected and added to 1.5 mL of cold phosphate buffer saline (pH 7.4, 0.05 M) to get a 10% (w/v) homogenate by using an automatic homogenizer (IKA T25 ULTRA-TURRAX Homogenizer). The homogenate was centrifuged for 20 min at a speed of 3000 rpm and 4 °C, and the supernatant was collected to measure oxidative stress markers, including malondialdehyde (MDA), glutathione (GSH), glutathione peroxidase (GPx), and superoxide dismutase (SOD). These markers were measured by the ELISA method, using commercially available kits from Shanghai Sunred Biological Technology (Shanghai, China).

### 2.4 Histopathological examination

#### 2.4.1 Transmission Electron Microscopy (TEM)

The left testis samples were cut into small pieces and processed for transmission electron microscopy (TEM) and light microscopy. For electron microscopy, tissues were fixed in 2.5% glutaraldehyde solution. After overnight fixation, tissues were washed in a phosphate buffer and kept for 2 h in 2% osmium tetroxide. Then, samples were subsequently dehydrated in a graded series of acetone solutions. After dehydration, the tissues were processed for embedding and sectioning. Ultrathin sections (70 nm) were stained first with uranyl acetate and then with lead citrate for electron microscopic examination as described by Bozzola and Russell (1999). The coded sections were then examined in a blinded manner (by a certified pathologist) for cellular changes.

#### 2.4.2 Hematoxylin and eosin staining

The paraffin-embedded tissue sections (4 µm thick) were deparaffinized in xylene (2 × 5 min) and rehydrated through a graded series of ethanol solutions (100%, 95%, and 70%; 2 min each). Then, sections were stained with hematoxylin (Harris) for 5–10 min and differentiated in 1% acid alcohol. Subsequently, they were subjected to the bluing step in alkaline water or Scott’s tap water substitute for 30–60 s and then rinsed for 5 min. The sections were counterstained with eosin Y (1–2 min), followed by dehydration through graded ethanol solutions (70%, 95%, and 100%), clearing in xylene (2 × 5 min), and mounting as described by Bancroft and Gamble (2013). The stained sections were evaluated in a blinded manner by a certified pathologist for the presence of lesions, including the atrophied tubules, presence of vacuolation, and altered germinal epithelia and basement membranes (Creasy et al., 2012).

#### 2.4.3 Periodic acid–Schiff (PAS) staining

After deparaffinization and rehydration, the sections were oxidized with 0.5%–1% periodic acid for 5–10 min and then rinsed. They were subsequently immersed in Schiff’s reagent for 10–15 min in the dark. Thereafter, the sections were washed in running tap water for approximately 5 min to develop the magenta coloration. The samples were then counterstained with Mayer’s hematoxylin for approximately 1 min before being blued. Finally, the sections were dehydrated, cleared in xylene, and mounted as described by Bancroft and Gamble (2013). The stained sections were coded and examined by a certified pathologist to evaluate the acrosomal components, spermatids, and basement membrane alterations (Creasy et al., 2012).

#### 2.4.4 TUNEL assay

Paraffin-embedded testis sections (4 µm thick) of testes were used for the assay. To assess apoptosis, terminal deoxynucleotidyl transferase dUTP nick end labeling (TUNEL) staining was performed using an *in situ* cell death detection kit with tetramethylrhodamine (TMR) red (catalog no. ab206386; Abcam, Cambridge, United Kingdom), following the manufacturer’s instructions. The coded stained sections were evaluated by a pathologist to detect apoptotic cells.

### 2.5 Molecular docking

The crystal structures of proteins such as cullin-3 (PDB ID: 1IUY), glutathione (PDB ID: 2WJU), GPx (PDB ID: 7U4K), SOD (PDB ID: 2JLP), IL-1β (PDB ID: 1HIB), TNF-α (PDB ID: 1CA4), IL-6 (PDB ID: 4J4L), Keap-1 (PDB ID: 7Q5H), NF-κB (PDB ID: 1ZK9), and Nrf2 (PDB ID: 7X5F) were obtained from the Protein Data Bank (PDB, https://www.pdb.org/pdb). A grid box with dimensions of 90 Å × 90 Å × 90 Å and a spacing of 0.45 Å was used during the docking procedure. BIOVIA Discovery Studio software was utilized to visualize the 3D structure of docking results (Jayaraman et al., 2024).

### 2.6 Molecular dynamics (MD)

Molecular dynamics (MD) simulations were performed using GROMACS to evaluate the protein (glutathione, GPx, cullin-3, and Il-1β)–ligand (AS IV and ATZ) interactions. The protein–ligand complexes were dissociated in PyMOL, and the corresponding topology files were generated in GROMACS. The AM-BER99SB-ILDN force field was used for the protein, while ACPYPE was used for the ligand. The combined topology was embedded within a dodecahedral box (1.0 nm buffer), solvated with the TIP3P water model, and neutralized with NaCl. Energy minimization was performed for 1,000 steps, followed by equilibration under constant volume (NVT, 300 ps at 300 K) and constant pressure (NPT, 500 ps at 1 bar) conditions, following standard GROMACS equilibration procedures. Subsequently, a 10 ns MD simulation was conducted, and the trajectory file (.xtc) was recorded for further analysis (Hertig et al., 2016; Velmurugan et al., 2025).

### 2.7 Statistical analysis

GraphPad Prism 7.0 software (GraphPad Software, San Diego, California, United States) was used for data analysis. The means of different experimental groups were compared using the one-way analysis of variance (ANOVA), followed by Bonferroni’s multiple comparison test to determine the mean differences between the groups. *p <0.05* was considered statistically significant. The study results were presented as the mean ± standard error of the mean (S.E.M).

## 3 Results

### 3.1 Effect of AS IV on changes in body weight and relative testicular weight (RTW)

In the present study, the ameliorative potential of AS IV on ATZ-induced testicular toxicity was evaluated in CD-1 mice after 21 days of ATZ treatment. The initial body weight, final body weight, body weight change, and absolute and relative testicular weights are presented in the Supplementary File (Supplementary Table S1). The mean percentage change in body weight (%) was significantly reduced in the ATZ-exposed group (*p < 0.001*) compared to the CON group. However, AS IV supplementation for 21 days significantly increased the body weight change (%) in the AS IV + ATZ group (*p < 0.01*) compared to the ATZ group. The mean absolute testicular weights (ATWs) in the experimental groups were as follows: CON group, 0.100 ± 0.02; ATZ group, 0.111 ± 0.003; AS IV group, 0.097 ± 0.03; ATZ + AS IV group, 0.102 ± 0.003. Although the ATW was high in the ATZ group compared to the CON group, it was not statistically significant (*p > 0.05*). The relative testicular weight (RTW; %) of the testis was calculated in all the experimental groups, and the mean values were as follows: CON group, 0.36 ± 0.01; ATZ group, 0.42 ± 0.011; AS IV group, 0.34 ± 0.02; ATZ + AS IV group, 0.37 ± 0.01. The mean RTW was significantly increased in the ATZ group (*p < 0.001*) compared to the CON group, whereas AS IV supplementation reduced the RTW in the ATZ-exposed group compared to the ATZ-alone group (*p < 0.01*).

### 3.2 Effect of AS IV supplementation on oxidative stress markers

**FIGURE 1:**
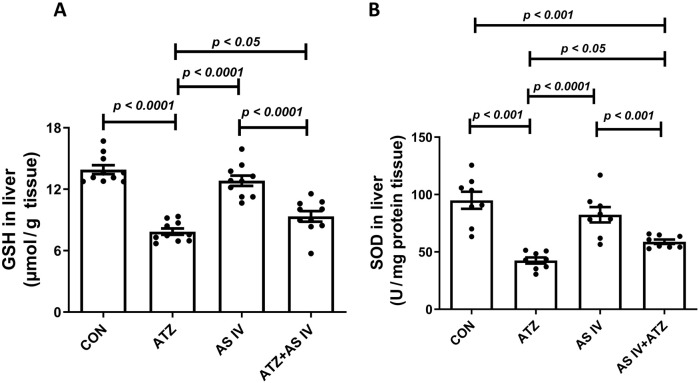
Effect of AS IV supplementation on glutathione (GSH) **(A)** and superoxide dismutase (SOD) **(B)** levels. Data are expressed as mean ± SEM. One-way ANOVA and Bonferroni’s multiple comparison test were used.

**FIGURE 2:**
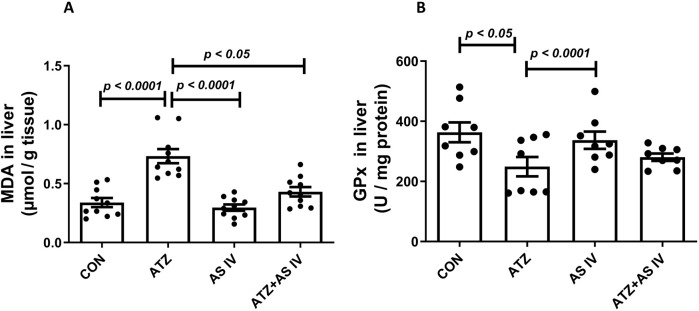
Effect of AS IV supplementation on MDA **(A)** and glutathione peroxidase (GPx) **(B)** levels. Data are expressed as mean ± SEM. One-way ANOVA and Bonferroni’s multiple comparison test were used.

The oxidative stress markers, including GSH, SOD, MDA, and GPx, were analyzed in liver homogenates. The ATZ exposure significantly reduced the GSH (*p < 0.001*) and SOD levels (*p < 0.001*) in the ATZ-exposed group compared to the CON group, whereas AS IV supplementation in ATZ-exposed mice significantly increased the GSH (*p < 0.05*) and SOD (*p < 0.05*) levels compared to the ATZ-alone group. Similarly, GPx activity was significantly reduced in the ATZ group (*p < 0.05*) compared to the CON group, while treatment with AS IV in ATZ-exposed mice increased the GPx activity, although this increase was not statistically significant compared to the ATZ group. MDA, the final product of lipid peroxidation, was significantly elevated in the ATZ group (*p < 0.001*) compared to the CON group. The MDA levels were significantly reduced with AS IV supplementation in ATZ-exposed mice compared to the ATZ-alone group (*p < 0.05*) (Figures 1, 2).

### 3.3 Effects of AS IV on the androgen-binding protein and testosterone levels

**FIGURE 3:**
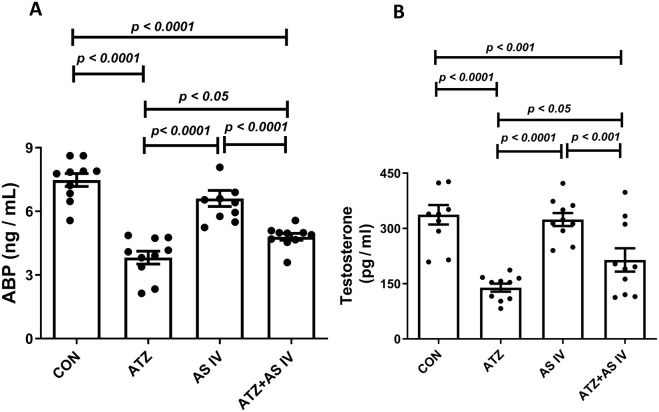
Effect of AS IV supplementation on androgen-binding protein **(A)** and testosterone **(B)** levels. Data are expressed as mean ± SEM. One-way ANOVA and Bonferroni’s multiple comparison test were used.

ATZ exposure significantly decreased the serum testosterone and androgen-binding protein (ABP) levels in the ATZ group (*p < 0.001*) compared to the CON group, whereas treatment with AS IV in ATZ-exposed mice significantly increased testosterone and ABP levels compared to the ATZ group (*p < 0.05*) (Figure 3).

### 3.4 Histopathological examination

**FIGURE 4:**
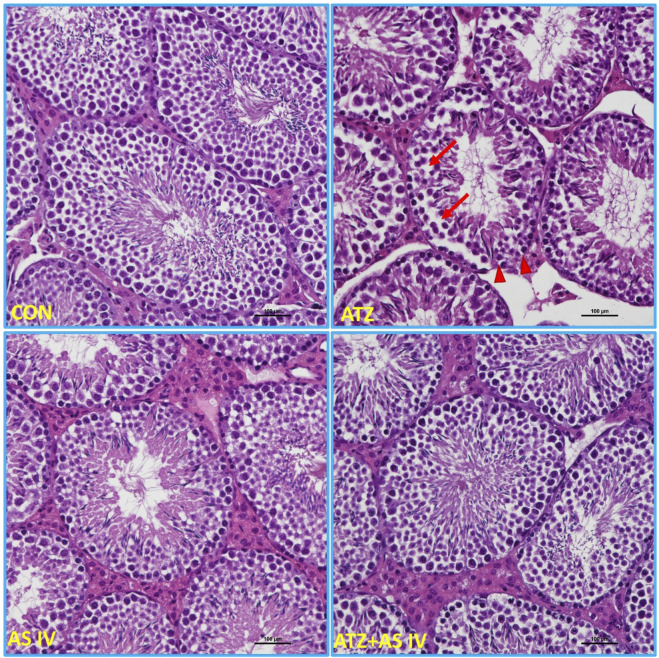
Light microscopy images of testis sections from experimental groups. The normal morphological features of seminiferous tubules were observed in the CON, AS IV, and ATA + AS IV groups. The testis sections of the ATZ-exposed mice showed sloughed and collapsed seminiferous epithelia. The presence of vacuolation and an altered basement membrane (arrowhead) was also observed. Widened interstitial areas with few spermatogonial lineage cells (arrows) were also noted in the ATZ-exposed mice. (Magnification 200×; H&E staining sections).

**FIGURE 5:**
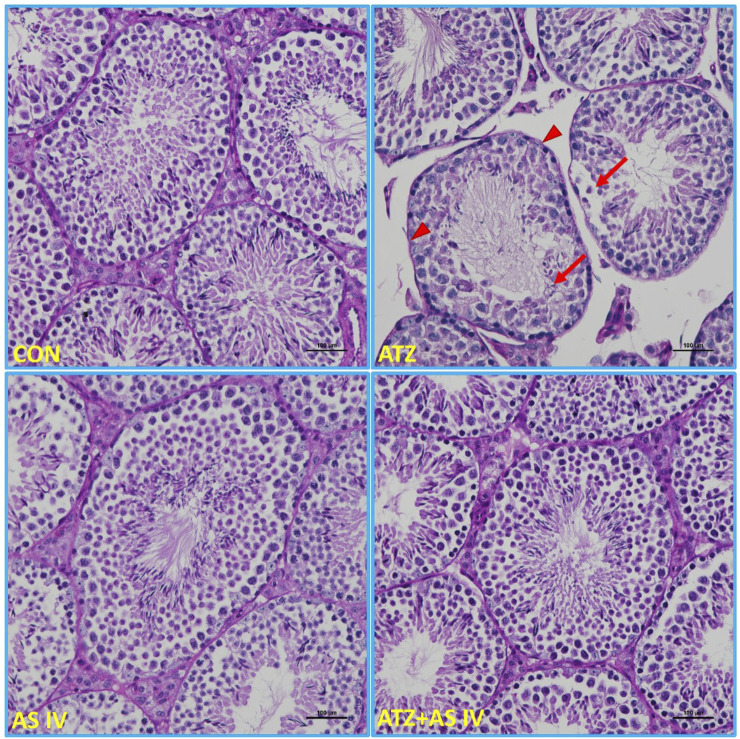
Light microscopy representative images of PAS-stained sections of testicular tissue from experimental groups. The intensely stained acrosomes of spermatids in the seminiferous tubules, basement membrane, and PAS-positive-stained material between the seminiferous tubules were observed in the CON, AS IV, and ATZ + AS IV group mice. Note the weak PAS-positive-stained basement membrane (arrowhead), spermatogonial cells, and the acrosomes of spermatids (arrows) in the ATZ-exposed mice. Treatment with AS IV in ATZ-exposed mice prevented the pathological changes in the testes. (Magnification 200×; PAS staining).

**FIGURE 6:**
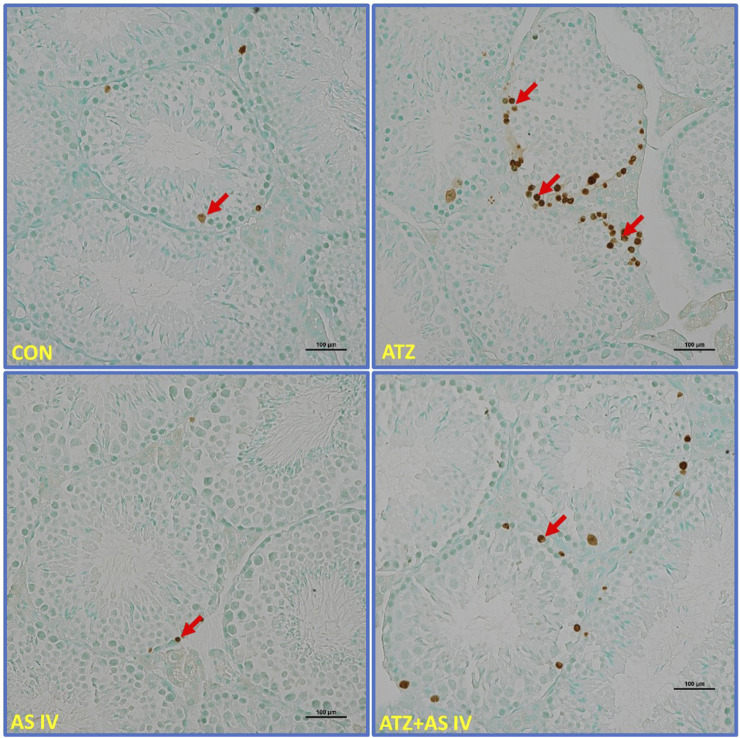
Light microscopy representative images of TUNEL-stained sections of testicular tissue from experimental groups. Note the extensively stained TUNEL-positive cells (arrows) in the seminiferous tubules of ATZ-exposed mice compared to other experimental groups.

Light microscopy revealed normal morphological features of seminiferous tubules in hematoxylin and eosin (H&E)- and PAS-stained sections of the CON and AS IV groups. Sloughed and collapsed seminiferous epithelium with vacuoles was observed in the ATZ-exposed mice. Widespread interstitial areas with few cells in the stratified epithelium, along with damaged basement membranes, were also noted in the ATZ-exposed group (Figures 4, 5). The TUNEL assay analysis showed extensive TUNEL-positive areas in the seminiferous tubules of the ATZ-exposed mice, whereas treatment with AS IV reduced these areas in ATZ-exposed mice (Figure 6).

### 3.5 Electron microscopy examination

**FIGURE 7:**
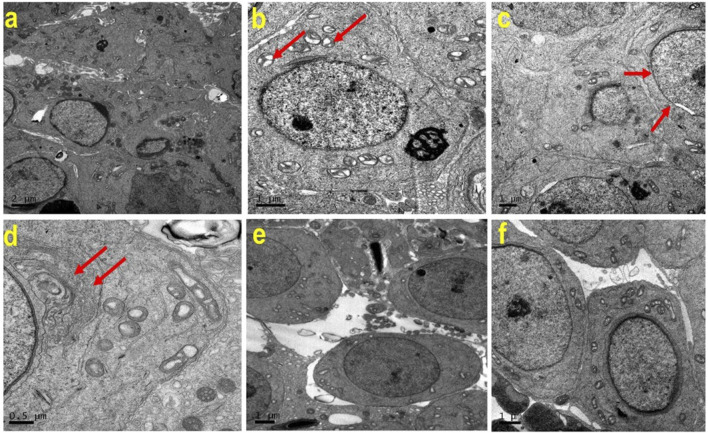
Representative transmission electron microscopy (TEM) images of testes from the experimental groups. Note the normal cellular features of Spermatogenic lineage cells in (a) (CON), (e) (AS IV group), and (f) (ATZ + AS IV group). Swollen mitochondria (b; red arrow), discontinuous nuclear membrane (c; red arrow), and dilated endoplasmic reticulum (d, red arrow) are seen in the testes of ATZ-exposed mice.

TEM was performed to examine the cellular ultrastructure of testicular tissue in the experimental groups. The analysis revealed ultrastructural changes in ATZ-exposed mice, including swollen mitochondria, discontinuous nuclear membranes (red arrow), and dilated endoplasmic reticulum. AS IV supplementation prevented these ATZ-induced alterations in the testicular tissue (Figure 7).

### 3.6 Docking results

**FIGURE 8:**
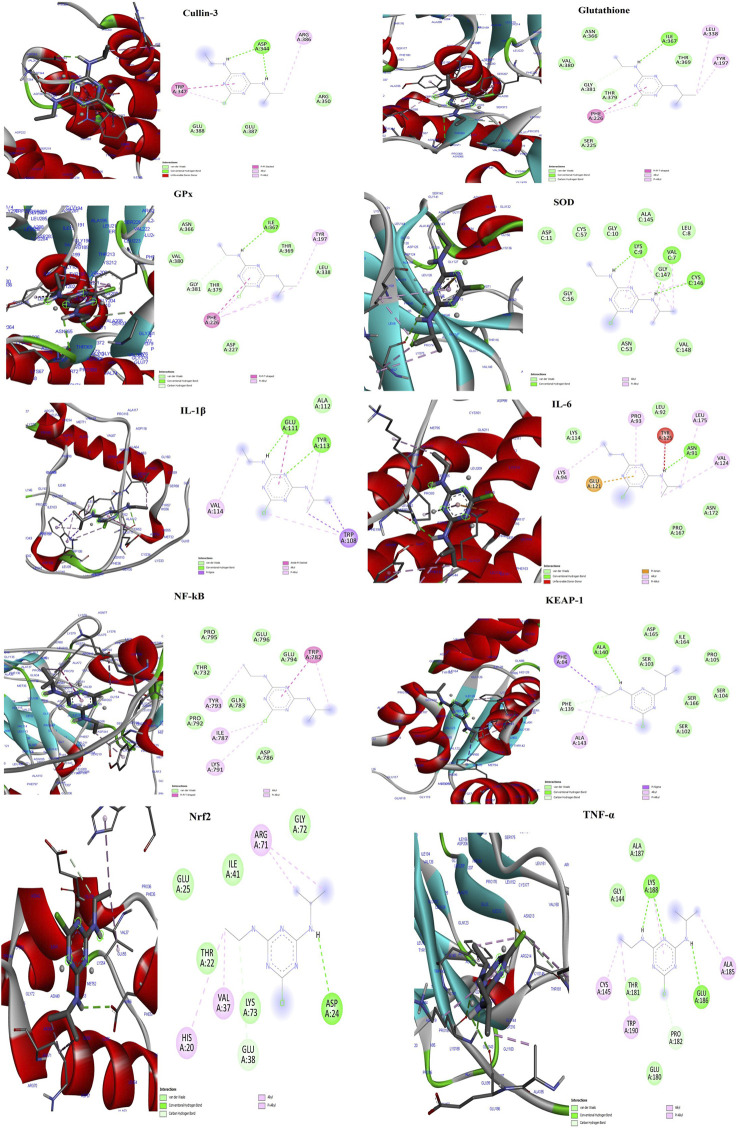
The molecular docking interactions of the AS IV compound with cullin-3, glutathione, GPx, SOD, IL-1β, TNF-α, IL-6, Keap-1, NF-κB, and Nrf2 proteins, highlighting binding modes, interacting residues, hydrogen bonds, and the protein–ligand conformations for each target.

**FIGURE 9:**
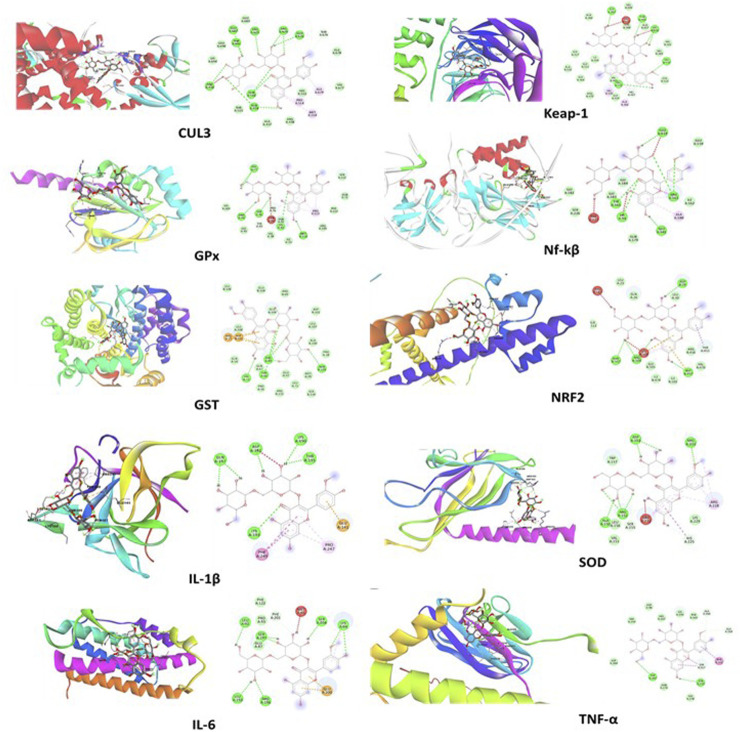
The visual representations illustrate docking interactions of the ATZ compound with cullin-3, glutathione, GPx, SOD, IL-1β, TNF-α, IL-6, Keap-1, NF-κB, and Nrf2 proteins, highlighting binding modes, interacting residues, hydrogen bonds, and the protein–ligand conformations for each target.

The docking study revealed that the ATZ and AS IV compounds interact with various target proteins such as cullin-3, glutathione, GPx, SOD, IL-1β, TNF-α, IL-6, Keap-1, NF-κB, and Nrf2, showing binding energies between −4.7 and −5.5 kcal/mol. Glutathione exhibited the strongest binding (−5.5 kcal/mol), with ILE A:367 as a key residue, while TNF-α and Nrf2 showed the weakest interactions (−4.7 kcal/mol). Notable interactions include strong stabilization with SOD (three hydrogen bonds) and potential modulation of antioxidants (SOD, Nrf2) and anti-inflammatory (IL-1β and TNF-α) pathways. These findings suggest therapeutic implications for ATZ’s modulation of oxidative stress and inflammation (Figures 8, 9; Tables 1, 2). Docking Table 1 summarizes key parameters, including binding energies (−4.7 to −5.5 kcal/mol), the number of hydrogen bonds (1–3), and interacting residues, demonstrating the compound’s moderate affinity and stabilization with specific protein targets. Docking Table 2 presents additional parameters, including binding energies (−6.3 to −9.2 kcal/mol), number of hydrogen bonds (1–7), and interacting residues, further illustrating the compound’s moderate affinity and stabilization with specific protein targets.

**TABLE 1:** Molecular docking results.

Protein	Binding energy (Kcal/mol)	No. of hydrogen bonds	Residues
	Atrazine compound		
Glutathione	−5.5	1	ILE A:367
GPx	−5.4	1	ILE A:367
Cullin-3	−4.9	1	ASP A:344
IL-1β	−5.1	2	GLU A:111, TYR A:113
IL-6	−4.8	1	ASN A:91
TNF-α	−4.7	2	LYS A:188, GLU A:186
SOD	−5	3	LYS C:9, VAL C:7, LYS C:146
Keap-1	−5	1	ALA A:140
NF-κB	−5	-	-
Nrf2	−4.7	1	ASP A:24

GPx, glutathione peroxidase; IL-1β, interleukin-1 beta; TNF-α, tumor necrosis factor alpha; SOD, superoxide dismutase; NF-κB, nuclear factor kappa-light-chain-enhancer of activated B cells; Nrf2, nuclear factor erythroid 2-related factor 2.

**TABLE 2:** Molecular docking analysis binding energy scores.

Protein	Binding energy (Kcal/mol)	No. of hydrogen bonds	Residues
	Astragaloside IV		
Glutathione	−9.2	4	VAL55, THR68, ARG69, ASN103
GPx	−7.2	5	HIS77, ARG44, SER46, LYS42, MET114
Cullin-3	−9.1	7	GLU687, ARG430, GLN516, GLN434, ARG629, GLU626, ARG625
IL-1β	−6.3	5	GLN197, ASP191, LYS190, THR195, LYS193
IL-6	−7.1	6	LEU193, ARG196, SER197, LEU92, SER204, LYS94
TNF-α	−7.1	2	TYR307, TYR215
SOD	−8.1	4	ASP153, ARG231, ARG152, ALA150
Keap-1	−8.9	5	GLY367, THR560, VAL608, VAL514, VAL512
NF-κB	−7.2	5	TYR165, LYS94, GLU181, ARG163, GLU119
Nrf2	−7.8	4	ASN507, ARG504, ASP457, ASP29

GPx, glutathione peroxidase; IL-1β, interleukin-1 beta; TNF-α, tumor necrosis factor alpha; SOD, superoxide dismutase; NF-κB, nuclear factor kappa-light-chain-enhancer of activated B cells; Nrf2, nuclear factor erythroid 2-related factor 2.

### 3.7 Molecular dynamics (MD)

**FIGURE 10:**
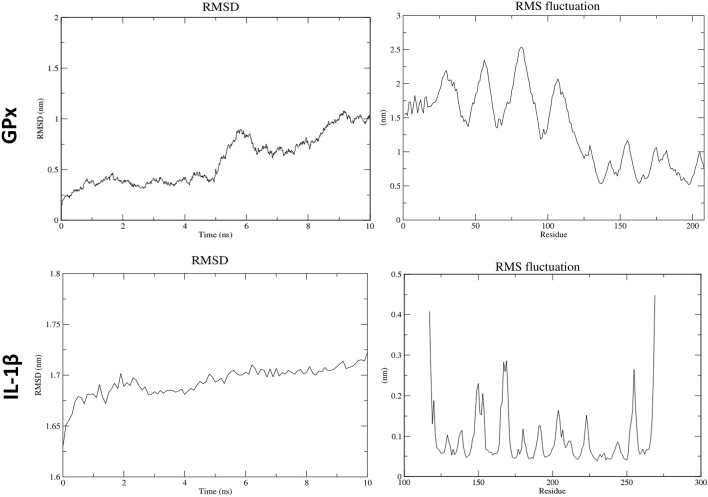
Molecular dynamics results for atrazine with GPx and IL-1β.

**FIGURE 11:**
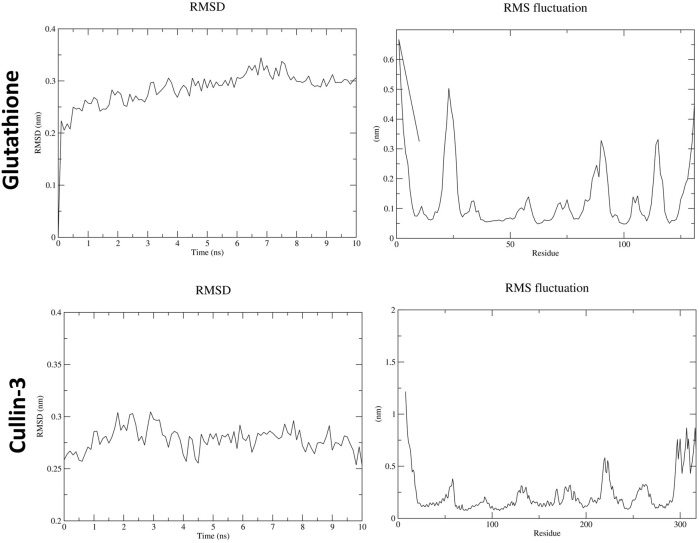
Molecular dynamics results for astragaloside IV with glutathione and cullin-3.

In the molecular dynamics (MD) simulation analysis, the periodic boundary conditions (PBC) were removed from the trajectory file (.xtc) at the beginning. Structural changes in ATZ–GPx and ATZ–IL-1β complexes, as well as in the AS IV–cullin-3 and AS IV–glutathione complexes, were analyzed using RMSD (root-mean-square deviation, structural deviation from the starting structure) and RMSF (root-mean-square fluctuation, residue fluctuations over time). RMSD graphs for GPx and IL-1β complexes with ATZ showed high peaks, which were attributed to dynamic loop regions. RMSF analysis revealed notable fluctuations in the N-terminal residues of both the GPx and IL-1β proteins. Collectively, RMSD and RMSF analyses confirmed the stability of all GPx–ATZ and IL-1β–ATZ complexes during the MD simulations (Figures 10, 11).

## 4 Discussion

The current study aimed to evaluate the ameliorative potential of AS IV against ATZ-induced male reproductive toxicity in mice, focusing on oxidative stress, inflammation, and histopathological alterations. The findings underscore AS IV’s promising protective effects, likely mediated through its antioxidant, anti-inflammatory, and anti-apoptotic properties. Exposure to ATZ significantly disrupted the redox balance in mice, as evidenced by reduced levels of GSH, SOD, and GPx, along with elevated MDA, a lipid peroxidation marker. These changes are consistent with prior reports of ATZ-induced oxidative stress in various tissues, including the testes, liver, and salivary glands (Victor-Costa et al., 2010). The observed oxidative damage reflects ATZ’s ability to generate reactive oxygen species (ROS), leading to impaired antioxidant defenses and cellular damage. A decreased body weight following chemical exposure, such as ATZ, indicates its role in systemic toxicity. An ATZ dose of 100 mg/kg body weight has been used in many experimental studies involving mice, rats, and rabbits to evaluate its toxic effects (Trentacoste et al., 2001; Scialli et al., 2014; Jin et al., 2015; Hao et al., 2016). Hence, in the present study, this dose was selected to study the toxic effects on the testis. In previous studies, a lower dose of AS IV (40 mg/kg) was reported to be effective against various experimentally induced disease conditions including cardiac hypertrophy, cardiomyopathy, liver damage, and renal toxicity (Ju et al., 2019; Lin et al., 2019; Hu et al., 2022; Zhang et al., 2023; Guo et al., 2024). Similar to these studies, in this study, AS IV at a lower dose was effective in mitigating the ATZ exposure-induced toxicity.

In the current study, body weight was significantly reduced in the ATZ-exposed mice, supporting similar reports in the literature in which ATZ doses above 50 mg/kg/body weight reduced body weight (Chang et al., 2021; Rotimi et al., 2024). The RTW was significantly increased in ATZ-exposed mice. An increased testicular weight could be attributed to the proportionate increase in the seminiferous tubules as well as the fluid-filled dilated lumens following the ATZ exposure. Similar observations of a transient increase in testicular weight were reported in the previous studies (Victor-Costa et al., 2010; Jin et al., 2013; Martins-Santos et al., 2017). The imbalance between reproductive hormones and their actions on the efferent ductules of the testis is one of the possible mechanisms behind the combined effects of atrophy and fluid accumulation (Victor-Costa et al., 2010).

Interestingly, supplementation with AS IV restored antioxidant enzyme activity and reduced MDA levels in ATZ-exposed mice. AS IV’s efficacy is likely attributed to its ability to scavenge free radicals, upregulate antioxidant pathways (e.g., Nrf2 activation), and enhance the activity of endogenous antioxidant enzymes. This aligns with previous studies demonstrating AS IV’s protective role in oxidative stress-related conditions, such as bisphenol A- and cadmium-induced toxicity (Essawy et al., 2021; Li et al., 2025). In the present study, ATZ exposure significantly reduced testosterone and ABP levels, which are critical for spermatogenesis and male reproductive health. ATZ’s endocrine-disrupting properties, including disruption of the hypothalamic–pituitary–gonadal axis and impairment of Leydig cell function, have been previously reported (Martins-Santos et al., 2017). Treatment with AS IV ameliorated these effects, partially restoring testosterone and ABP levels. This indicates AS IV’s potential to mitigate endocrine disruption, likely through anti-inflammatory and antioxidative mechanisms that protect Leydig cell function and the blood–testis barrier.

Histopathological examination revealed severe testicular damage in ATZ-exposed mice, including sloughing of the seminiferous epithelia, vacuolization, and altered basement membranes, along with increased apoptotic areas in spermatogonia cells in the seminiferous tubules. TEM examination revealed swollen mitochondria, dilated endoplasmic reticulum, and altered nuclear membranes. These findings are consistent with the ATZ-induced apoptosis, disrupted spermatogenesis, and structural damage reported in earlier studies (Durand et al., 2020; Genovese et al., 2021). Remarkably, treatment with AS IV mitigated these pathological changes, preserving the structural integrity of seminiferous tubules and cellular components. This supports AS IV’s role in maintaining testicular architecture, likely by attenuating oxidative damage, lipid peroxidation, and inflammation. Docking studies provided valuable insights into the molecular interactions of ATZ with key proteins involved in oxidative stress and inflammation. ATZ exhibited moderate binding affinities (−4.7 to −5.5 kcal/mol) with targets such as glutathione, GPx, SOD, and inflammatory mediators (IL-1β, TNF-α, and NF-κB). Among these, glutathione showed the strongest interaction (−5.5 kcal/mol), highlighting its role in ATZ-induced oxidative stress. AS IV displayed strong binding affinities with key molecular targets involved in cellular redox balance and protein degradation pathways. Specifically, it demonstrated a binding affinity of −9.2 kcal/mol with glutathione, suggesting a potential role in modulating oxidative stress. Additionally, it interacts with cullin-3 at −9.1 kcal/mol, which may indicate an influence on ubiquitin-mediated protein degradation. Its binding affinity with keap-1 (−8.9 kcal/mol) highlights its possible involvement in regulating the Nrf2 signaling pathway, a crucial mechanism for cellular defense against oxidative damage. These interactions collectively suggest that AS IV may exert significant therapeutic effects by modulating oxidative stress, protein homeostasis, and cellular protective mechanisms. Molecular dynamics (MD) simulations capture atomic-level movements over time, revealing dynamic behavior, binding affinities, and structural flexibility. This helps optimize drug design, predict binding modes, and assess ligand-induced protein conformational changes (De Vivo et al., 2016; Hertig et al., 2016). In MD simulation, both RMSD and RMSF analyses validated the stability of GPx– and IL-1β–ATZ complexes and glutathione and cullin-3–AS IV complexes, with significant fluctuations identified in the loop regions as well as in the initial protein residues.

Importantly, AS IV’s protective effects may stem from its ability to modulate key signaling pathways, as previously demonstrated in models of oxidative stress and inflammation. Stabilization of SOD (via hydrogen bonding) and modulation of Nrf2 and NF-κB pathways suggest AS IV’s dual antioxidant and anti-inflammatory actions. These findings align with its reported ability to mitigate cadmium-induced spermatogenesis disruption and other toxicological effects (Ning et al., 2022; Li et al., 2025). The study provides compelling evidence that AS IV ameliorates ATZ-induced male reproductive toxicity by enhancing antioxidant defenses, reducing inflammation, and preserving histological integrity. While the docking results highlight ATZ’s molecular interactions with target proteins, future studies are needed to investigate the direct molecular mechanisms underlying AS IV’s protective effects. AS IV emerges as a promising natural compound for mitigating ATZ-induced male reproductive toxicity, with potential therapeutic implications for addressing the harmful effects of herbicide exposure. By modulating oxidative stress and inflammation, AS IV demonstrates significant potential for preserving male reproductive health, warranting further exploration into its molecular mechanisms and broader applications. Overall, the study highlights AS IV’s efficacy in protecting against ATZ-induced male reproductive damage by enhancing antioxidant defenses, reducing inflammation, and preserving testicular architecture, supporting its potential as a therapeutic agent for combating herbicide-induced reproductive toxicity.

The present study provides strong evidence that AS IV mitigates ATZ exposure-induced male reproductive toxicity. However, certain limitations should be considered when interpreting these results. The ATZ dose (100 mg/kg/day) used in this study, although consistent with previous toxicological research, represents a relatively high exposure compared with environmentally relevant levels in humans. Therefore, some observed effects may reflect non-specific systemic toxicity, such as body weight reduction or metabolic stress, rather than endocrine-mediated mechanisms. The decrease in body weight in ATZ-exposed mice may have contributed indirectly to reproductive impairment by influencing hormonal synthesis and spermatogenesis. Moreover, the short exposure duration (21 days) was sufficient to demonstrate testicular and hormonal changes but may not fully capture the chronic or cumulative effects of prolonged low-dose ATZ exposure. Species differences in metabolism, endocrine regulation, and xenobiotic clearance further limit direct extrapolation of these findings to humans. While the results highlight the ameliorative potential of AS IV, additional studies using human cell models or pharmacokinetic simulations are necessary to confirm its translational relevance.

This study primarily focused on oxidative stress and histopathological alterations, without assessing the hypothalamic–pituitary hormonal regulation or pharmacokinetic behavior of AS IV. Considering AS IV’s limited solubility and absorption, evaluating its bioavailability and tissue distribution would improve understanding of its therapeutic window. In conclusion, although AS IV shows significant protective efficacy against ATZ-induced reproductive toxicity, data interpretation should consider the high-dose exposure paradigm, possible systemic effects, species differences, and limited endocrine and pharmacokinetic profiling. Future studies with lower, environmentally relevant doses and extended mechanistic analyses are warranted to strengthen the translational potential of AS IV.

## 5 Conclusion

ATZ exposure for 21 days impaired testicular function and was associated with structural alterations and oxidative imbalance. AS IV simultaneous supplementation significantly improved antioxidant status, partially restored the reproductive hormones, and alleviated the structural damage in ATZ-exposed mice. The docking and molecular dynamics results further supported the potential interactions of AS IV with oxidative and inflammatory pathway proteins, suggesting its modulatory role in cellular protection. While these findings indicate a strong association between oxidative stress and ATZ-induced testicular toxicity, further mechanistic studies are warranted to confirm the direct causal pathways. Overall, AS IV appears to be a promising natural compound for mitigating ATZ-associated male reproductive toxicity.

## Data Availability

The raw data supporting the conclusions of this article will be made available by the authors, without undue reservation.
