# Supplementary material for: Astragaloside IV ameliorates atrazine-induced male reproductive toxicity: an in vivo and in silico analysis
Source: Front Toxicol. 2025 Dec 22;7:1692518. doi: 10.3389/ftox.2025.1692518 (PMC12766972; doi:10.3389/ftox.2025.1692518)
Supplement: Supplementary file 1 [file Table1.doc]

Table1. Effect of Astraloside IV (AS IV) treatment on body weight and testes in rats with atrazine (ATZ)-induced male reproductive toxicity.

| **ATZ + AS IV** | **AS IV** | **ATZ** | **Control** | **Parameters/**  **Treatment** |
| --- | --- | --- | --- | --- |
| 27.22 ± 0.20 | 27.71 ± 0.31 | 27.37 ± 0.42 | 27.63 ± 0.58 | **Baseline body weight (g)** |
| 27.65 ± 0.29 | 28.44 ± 0.40**^b^** | 26.16 ±0.35**^a^** | 28.24 ± 0.50 | **Final body weight (g)** |
| 1.57± 0.67**^b^** | 2.63 ± 0.60 **^b^** | -4.36 ± 0.89**^a^** | 2.29 ± 0.82 | **Body weight change (%)** |
| 0.102 ± 0.003 | 0.097 ± 0.003**^b^** | 0.111± 0.003 | 0.100 ± 0.002 | **Total testes weight (g)** |
| 0.37± 0.011**^b^** | 0.34 ± 0.012 **^b^** | 0.42 ± 0.010**^a^** | 0.36 ± 0.010 | **Relative testes weight (%)** |

Values in the table are means ± SEM (n = 10).

Differences between the groups were assessed by one-way analysis of variance (ANOVA) followed by Bonferroni’s multiple comparison test, Where *P* < 0.05.

**^a^** denotes significance of Control group vs. different groups. **^b^** denotes significance of ATZ alone group vs. ATZ treated other groups.
